# Enhancing multilevel inverter performance through open-end winding motors and SVPWM series compensation

**DOI:** 10.1038/s41598-025-19849-z

**Published:** 2025-10-15

**Authors:** Surasmi Nalinakshan Latha, Suresh Kumar Egeriose, Shiny Gopinathan

**Affiliations:** 1 Power Electronics Research Laboratory, College of Engineering Trivandrum, Thiruananthapuram, Kerala India; 2 Department of Electronic and Communication Engineering, College of Engineering Trivandrum, Thiruvananthapuram, Kerala India; 3 Dean (Electronics and Electrical Engineering Department), Saintgits College of Engineering, Kottayam, Kerala India; 4 APJ Abdul Kalam Technological University, Thiruvananthapuram, 695016 Kerala India; 5 Department of Electrical and Electronics Engineering, Mar Baselios College of Engineering and Technology (Autonomous), Mar Ivanios Vidyanagar, Nalanchira, Thiruvananthapuram, 695015 Kerala India

**Keywords:** Multilevel inverter, Open-end winding induction motor, Space vector pulse width modulation, Series compensation, Total harmonic distortion, Energy science and technology, Engineering

## Abstract

This study proposes a unique control approach to improve the power quality of a multilevel inverter architecture based on an Open-End Winding Induction Motor (OEWIM) drive system. To obtain an open-end induction motor, two different voltage-source inverters are fed from both ends of the star-connected primary winding. The OEWIM is supplied from one end by a main three-level inverter that is created by cascading dual two-level inverters which are controlled using space-vector modulation (SVM). To offset the harmonics generated by the three-level inverter, a supplemental inverter that operates as a series compensator is connected to the other end of the OEWIM. An active filtering control method is utilized to govern the supplemental two-level inverter to reduce the voltage harmonic distortions brought on by the three-level inverter. The strategy leverages the inherent advantages of OEWIMs, such as enhanced fault tolerance and reduced common-mode voltage, making the system suitable for high-performance industrial applications. Detailed analysis of the inverter’s switching patterns, simulations, and experimental validation demonstrate significant improvements in power quality for different modulation indices. As the motor voltage and current waveforms’ harmonic distortions diminish, the drive’s efficiency will rise.

## Introduction

The demand for high-performance motor drive systems with superior power quality and efficiency has been growing steadily across various industrial applications. Induction motors, particularly Open-End Winding Induction Motors (OEWIM), have gained significant attention due to their inherent advantages, including improved fault tolerance, higher dynamic performance, and the capability to operate at extended voltage levels. The open-end winding configuration involves connecting Voltage Source Inverters (VSIs) at both ends of the motor windings, creating opportunities for advanced control and multilevel operation without requiring complex hardware.

Open-End Winding Induction Motor (OEWIM) based multilevel inverters have emerged as a highly efficient solution for applications requiring enhanced power quality, reduced harmonic distortion, and robust fault tolerance. Space vector PWM (SVPWM) has been extended into hybrid, clamping, and decoupled forms to suppress Total Harmonic Distortion (THD), enlarge the linear modulation range, and achieve better utilization of the DC link. Asymmetric control strategies and decoupled SVPWM were experimentally validated to demonstrate their effectiveness in reducing Common Mode Voltage (CMV) and improving dynamic response^1,2^. Furthermore, hybrid clamping and multi-vector SVPWM methods have been proposed to generate four-level operation from two-level inverters, striking a balance between harmonic reduction and DC-link voltage stress^3,4^.

High-frequency noise, bearing currents, and electromagnetic interference remain challenges in OEWIM systems. Recent contributions show that selective vector sequence design and discontinuous PWM patterns can significantly reduce CMV without compromising drive performance^5^. Comparative analyses indicate that dual-inverter OEWIM drives can achieve similar or superior quality to conventional three-level inverters while retaining the flexibility of separate DC sources^6^. Researchers have investigated fault-tolerant nine-level inverter structures, as well as T-type inverter-fed OEWIM drives for electric vehicle applications^7,8^. These approaches not only increase the fault resilience of the system but also reduce inverter rating requirements. In addition, OEWIM drives are being increasingly studied in renewable energy and hybrid source applications, where two separate inverters can be fed from independent sources such as PV arrays, batteries, or fuel cells. This makes OEWIM particularly attractive in traction and standalone energy systems^9,10^.

Recent trends also include AI-based and predictive control strategies. For instance, artificial neural network (ANN)-assisted fault detection has been integrated into OEWIM drives to enhance reliability under abnormal conditions^11^. Similarly, model predictive control (MPC)-based schemes have been applied to optimize switching decisions in real time, achieving better transient performance and reduced harmonic distortion compared to conventional PWM^12^. An advanced approach to enhancing power quality in grid-connected photovoltaic systems through the combined use of a hybrid energy storage system and a three-phase ten-switch (H10) inverter is proposed in^13^. The hybrid energy storage system, integrating both batteries and supercapacitors, improves system stability by ensuring sustained energy supply alongside rapid transient power support. The H10 inverter, operated with a space vector pulse width modulation (SVPWM) strategy, effectively minimizes common-mode voltage (CMV) fluctuations and voltage stresses, achieving performance comparable to three-level inverters while utilizing fewer components.

Control strategies such as Space Vector Pulse width Modulation (SVPWM)^14,15^, Model Predictive Control (MPC)^16–18^, and phase-shifted PWM^19^ play a critical role in optimizing system performance. SVPWM is widely utilized for its ability to generate optimal switching sequences, while MPC has shown promise in dynamic performance enhancement and fault tolerance^20^. Advanced fault-tolerant control strategies, including redundancy allocation and fault diagnosis mechanisms, have been proposed to ensure continuous operation during switch or phase failures^21,22^. Harmonic mitigation remains a central challenge in OEWIM-based systems, with active harmonic compensation techniques^23^, phase disposition modulation^24^, and resonant controllers^25^ emerging as effective solutions to address this issue. Recent research highlights the development of hybrid multilevel inverter topologies that combine the strengths of various configurations to achieve higher efficiency and reliability^26,27^.

A SVPWM strategy tailored for a hybrid-level three-phase inverter to effectively mitigate common-mode voltage (CMV) is discussed in^28^. The method achieves reduced CMV and switching losses while maintaining output quality comparable to conventional multilevel inverters. Simulation and experimental validation confirm its effectiveness, making it suitable for reliable and efficient industrial and renewable energy applications.A future trends in OEWIM-based multilevel inverters include their integration into smart grids, electric vehicles, and high-performance industrial drives, where advanced digital control techniques and renewable energy compatibility are expected to drive further innovation^29,30^. Power quality improvement in Multilevel inverters (MLIs) is a critical area of research, as MLIs provide superior performance in various applications like motor drives, renewable energy integration, and industrial systems. Multilevel inverters produce high-quality output waveforms with reduced harmonic distortion compared to traditional two-level inverters, making them essential for systems that require stable and reliable power supply.

One of the most significant methods to enhance power quality in MLIs is through advanced modulation techniques. Space Vector Pulse Width Modulation (SVPWM) and Phase-Shifted Pulse Width Modulation (PSPWM) are two popular approaches that help in reducing the Total Harmonic Distortion (THD). SVPWM is particularly effective in generating sinusoidal waveforms, which improves the overall quality of the output voltage by spreading the harmonics more evenly across higher frequencies, thus minimizing low-order harmonics that typically cause power quality issues. PWM allows for better control over the switching sequence of the inverter, contributing to lower THD levels for medium voltage AC motor driving applications. In order to lessen phase voltage distortion in multilayer inverters, an open end winding machine configuration and an appropriate control technique are provided in^31^. One side of the machine receives active power from a primary multilevel inverter, while the other side is filtered by an auxiliary two-level inverter. For a dual bridge inverter and a dual three-phase induction motor drive with open stator phase windings, a single DC voltage supply is advised in^32^. A decoupled SVPWM architecture is used to control voltage distortion.

Without changing the power circuit architecture or the device voltage ratings, an enhanced SVPWM scheme that outperforms the Decoupled SVPWM scheme in terms of harmonic performance is described in^33^. In order to simultaneously supply regulated power to frequency-insensitive ac-loads and dc-link loads, an Induction Generator (IG) system with open-end stator windings is recommended^34^. This system controls the capacitor-fed VSI to supply the reactive power required by the IG while maintaining the dc-link and ac load voltages at the appropriate levels.

This paper presents a method for enhancing a three-level inverter’s harmonic performance with an OEWIM drive. Unlike a conventional drive, which shares the motor’s power requirements between two separate Voltage Source Inverters (VSIs), an induction motor has a main three-level inverter at one end and a supplemental VSI that acts as a dynamic voltage restorer at the other end. Two two-level inverters are connected with half DC-bus voltage to create the main inverter. The SVPWM method is employed to run both inverters. The difference between the main three-level inverter’s voltage waveform and the basic reference-voltage waveform is the output voltage waveform produced by the supplemental VSI. Consequently, the effective motor phase voltage and current harmonics are greatly decreased and the drive efficiency increases.

## Open end winding induction-motor configuration

**Fig. 1:**
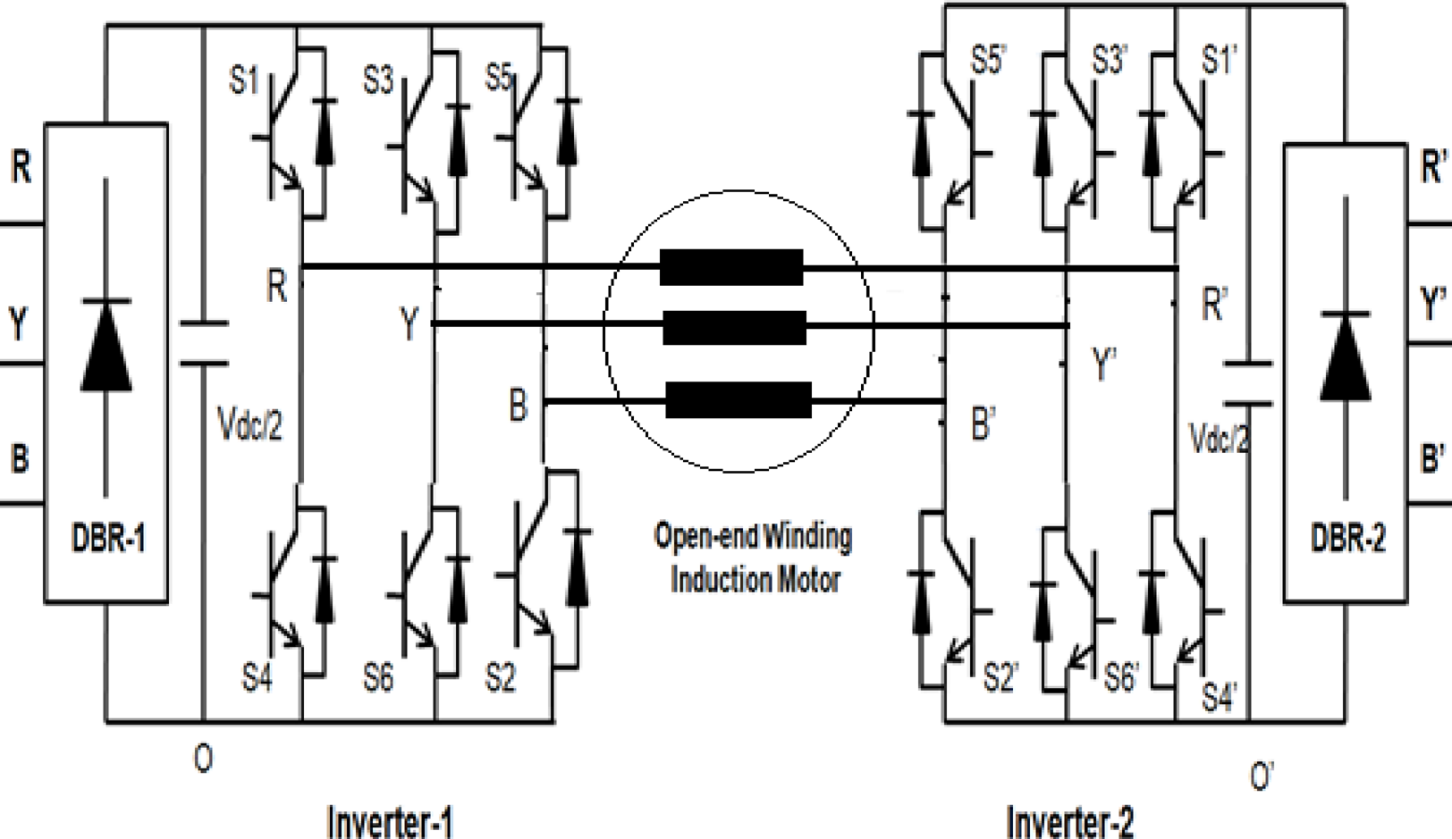
Circuit diagram of OEWIM drive.

Figure 1 shows a schematic of a basic Open End Winding Induction Motor (OEWIM) drive circuit. An open end winding induction motor is produced by opening the neutral point of the stator windings that are connected to the star and using two separate inverters to feed on either side. Since the two VSIs share the active power required by the motor under the basic OEWIM design, they are typically the same or comparable size and are powered by two independent power sources. Both inverters give the AC motor the same amount of active power. The common-mode voltage and harmonic distortion are reduced by using a synchronous optimum pulse width modulation.

### Cascaded three-level inverter

Three level inverter designs can also be achieved by cascading two VSIs. In^3^, a space vector pulse width modulation approach for a three-level inverter that is implemented by cascading two two-level inverters is proposed. There are 64 space vector combinations spread across 19 space vector locations in the cascaded topology’s space vector diagram. In contrast to the traditional Neutral Point Clamped multilevel inverter, the cascaded architecture does away with clamping diodes and neutral point variations.

**Fig. 2:**
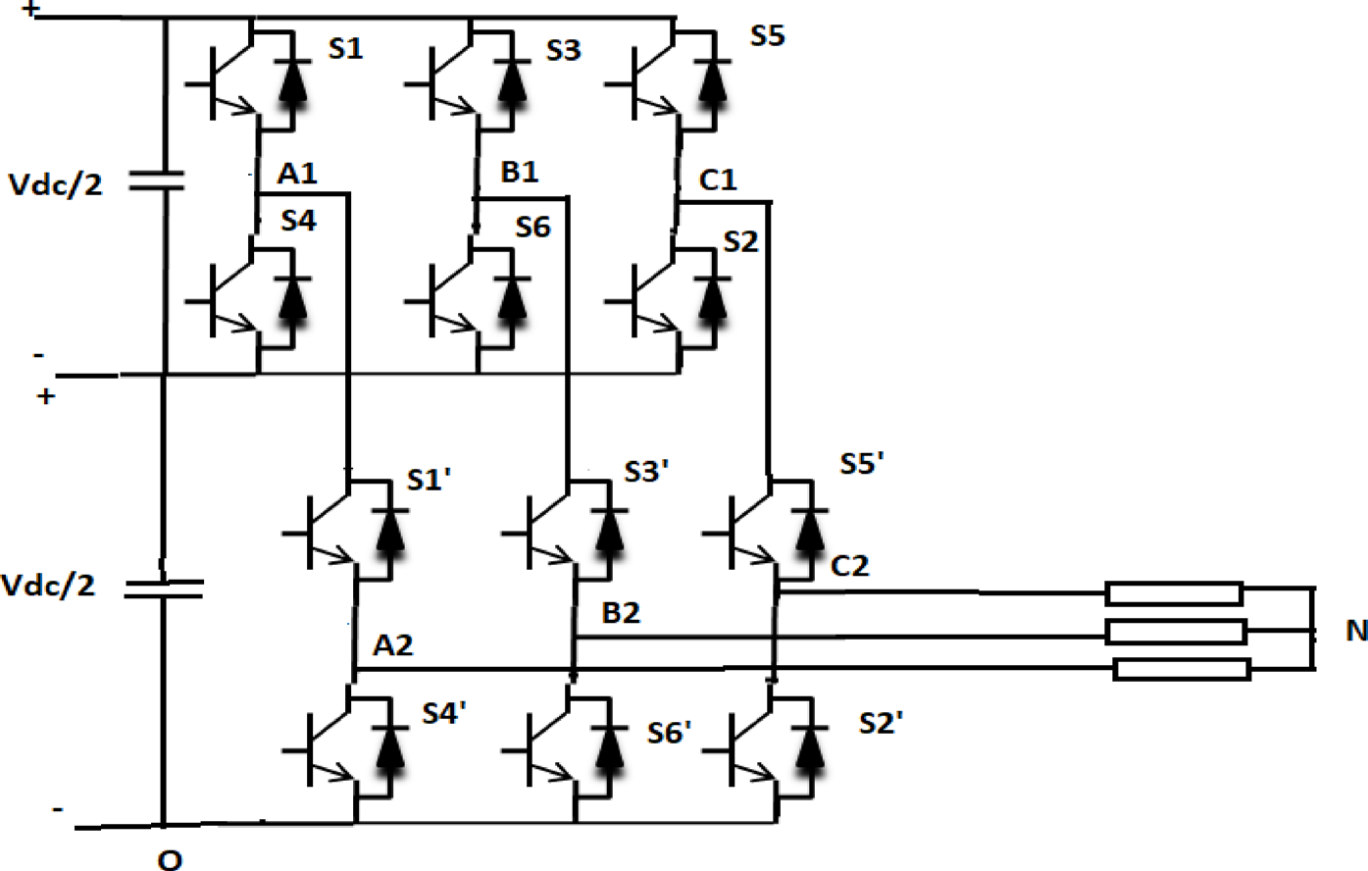
Three level inverter realized with cascaded connection of dual two level inverters.

By cascading two two-level inverters together, a three-level inverter system is achieved (Fig. 2). The top DC link rail of the corresponding phases of Inverter-2 is linked to the three output phase points of Inverter-1 in a cascaded three level inverter configuration. The output points of Inverter-2 are connected to the three-phase induction motor. Each inverter’s DC link voltage is Vdc⁄2. The output pole voltages of inverter-1 are V_A1O_, V_B1O_, and V_C1O_ when DC-link neutral is set to O. Likewise, inverter-2’s output pole voltages are V_A2O_, V_B2O_, and V_C2O_. The switches’ condition and the actual voltage levels for A-phase are displayed in Table 1.

**Table 1:** Inverter switch states, pole voltages and output voltage level.

Switch states		Inverter output pole voltage VA2O	Voltage level
S1	S1’		
OFF	OFF	0	0
OFF	ON	$$\:\raisebox{1ex}{${V}_{dc}$}\!\left/\:\!\raisebox{-1ex}{$2$}\right.$$	1
ON	OFF	0	0
ON	ON	V_dc_	2

**Fig. 3:**
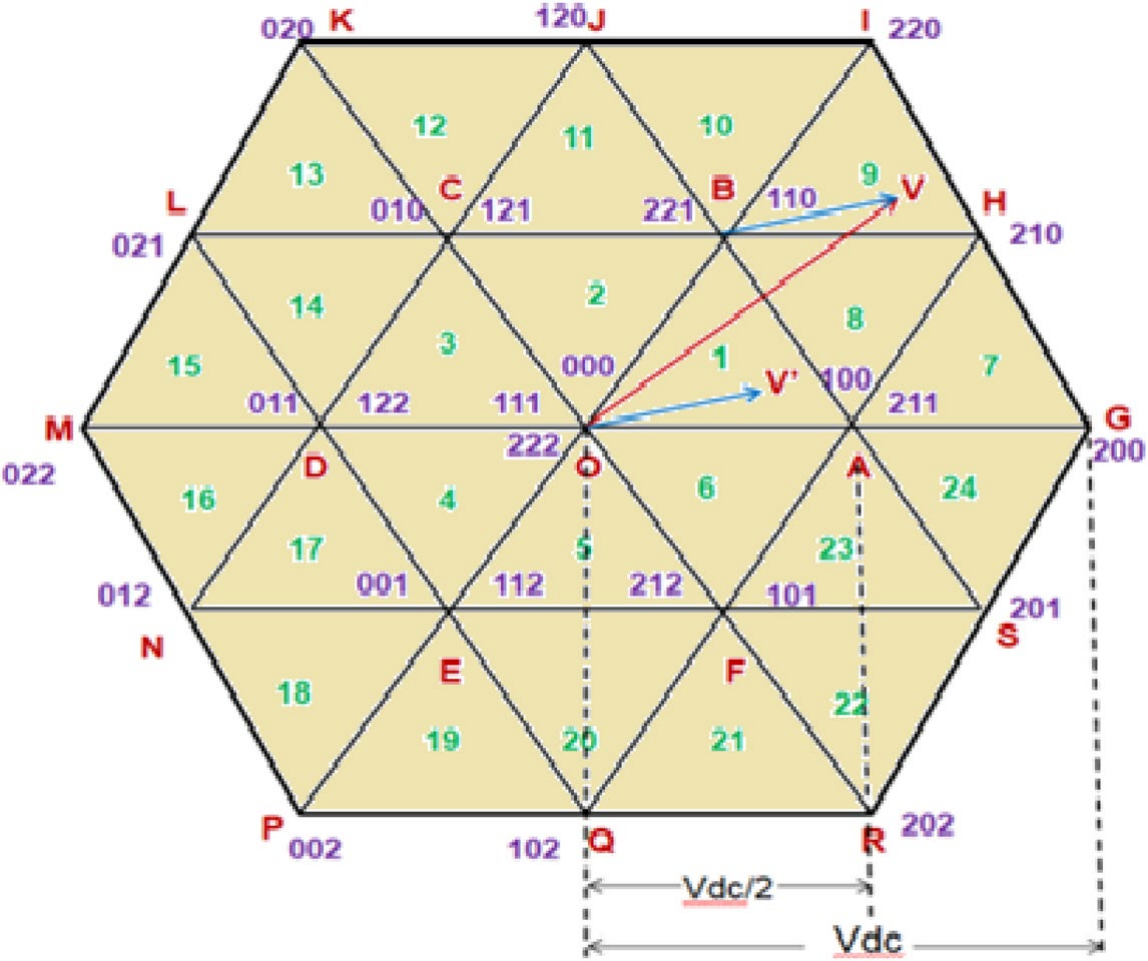
Three level inverter space vector diagram.

Figure 3 displays the three-level space vector diagram of the cascaded arrangement, which consists of 19 space vector locations with 24 sectors and 64 switching states. To generate the switching vector combinations and calculate the switching time period, the reference space vector is mapped to the inner sub hexagon. By comparing the instantaneous amplitudes of the three phase reference voltages Va, Vb, and Vc, one can determine the subhexagon center close to the reference vector’s tip. Using appropriate coordinate transformation, the center of the outer subhex is moved in mapping so that it coincides with the center O (000) of the inner subhex^3^. The standard formulae for a two-level inverter can now be used to calculate the switching vectors’ duration.

In Fig. 3 the reference space vector OV is shown in the space-vector diagram. The vectors OB and BV can be thought of as the outcome of the reference space vector OV. Here, the opposite vector of BV is used to switch the second inverter, while the base vector OB is used to switch the first inverter. The inner hexagon is initially mapped to the vector BV, which is represented as OV’. For inverters, the switching duration is calculated as given in^35^.

### Proposed OEWIM with series compensation

**Fig. 4:**
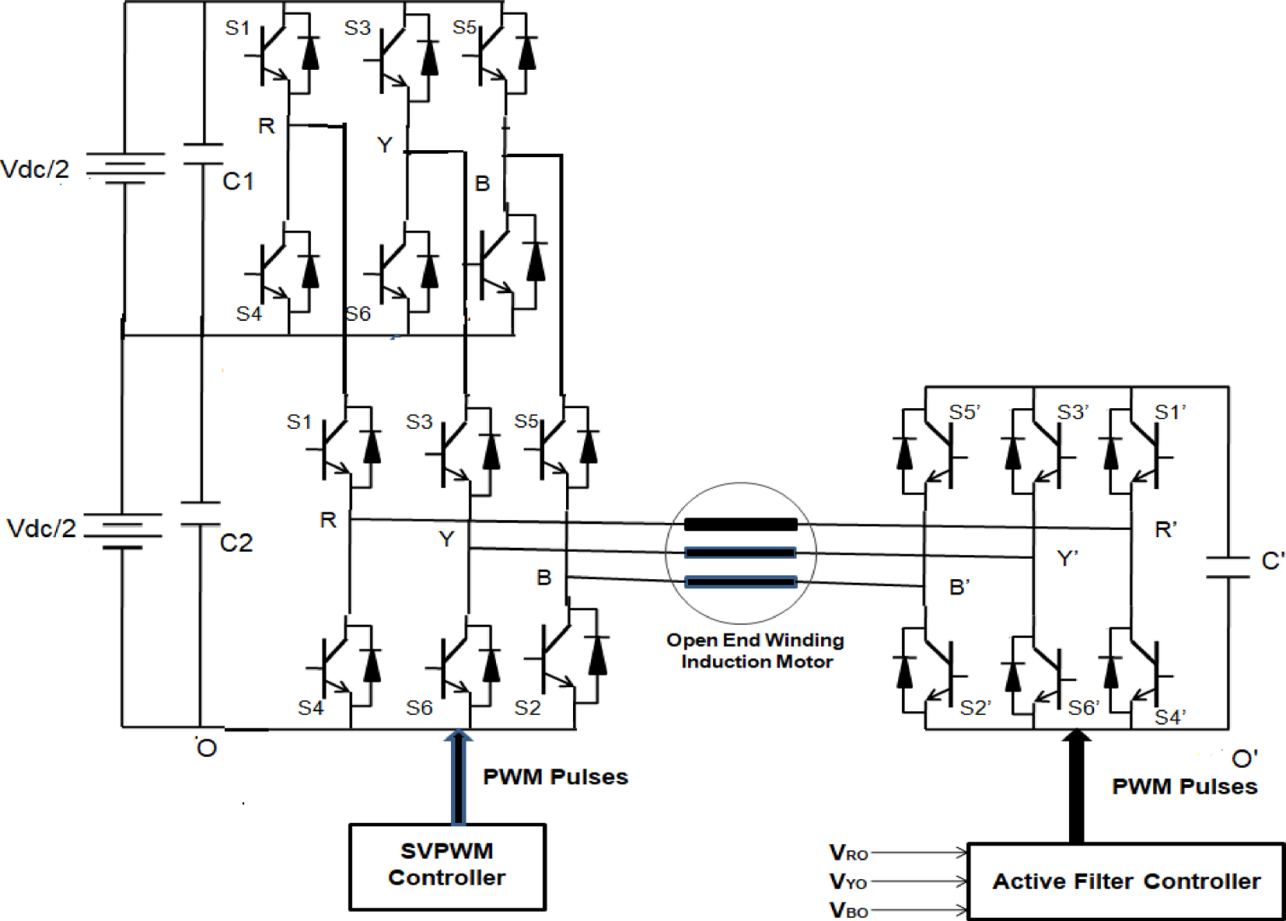
Power circuit of the proposed topology.

Figure 4 displays the proposed system’s block diagram. This analyzes the circuit topology of a three-level open-end winding induction motor drive. With just one DC voltage source at the converter’s primary side, this design makes use of two inverters. The suggested method uses a secondary two-level inverter as an active filter and a primary inverter to supply all of the active power. The latter can be provided by a floating capacitor, which eliminates the need for an extra independent power source and provides zero average active power. The secondary unit functions as a series active filter to offset the harmonic distortion, while the primary inverter is controlled by a highly efficient Space Vector Pulse Width Modulation (SVPWM). The torque ripple can be decreased and the drive efficiency raised by lowering the phase voltage harmonic content.

The suggested open end winding induction motor drive architecture, as illustrated in Fig. 4, consists of a supplemental two-level inverter serving as an active power filter and a primary inverter serving as the main power unit. To avoid zero sequence currents, the DC-buses of the two inverters are not directly connected to one another. The open winding machine can be powered by a floating capacitor since the two-level inverter should ideally provide zero average active power. The pulses for the secondary two-level inverter are obtained using the active filter control technique, which removes distortions from the first inverter output waveform. The SVPWM technique is used to generate the pulses from the primary inverter. Because of the control algorithm, the secondary inverter will produce an output voltage that is equal to the harmonic distortions produced by the primary inverter. The motor phase voltage that results from an OEWIM is the difference between the primary and secondary inverter voltages. As a result, the effective motor voltage harmonics will drop.

#### Series active filter algorithm

**Fig. 5:**
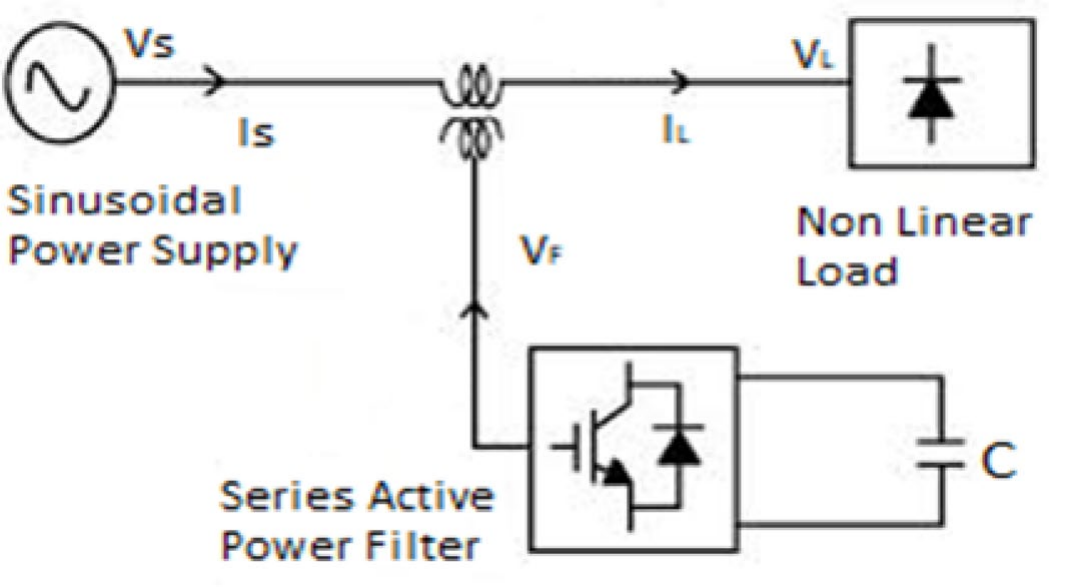
Series active power filter.

Series active power filters are active power filters that are linked in series with the load. This functions as an isolator rather than a harmonic generator. Voltage sources that can be controlled are series active power filters. This is adjusted to pull or inject a compensating voltage into or out of the supply, canceling harmonics of the load-side voltage. The series active power filter concept is shown in Figure 5.

**Fig. 6:**
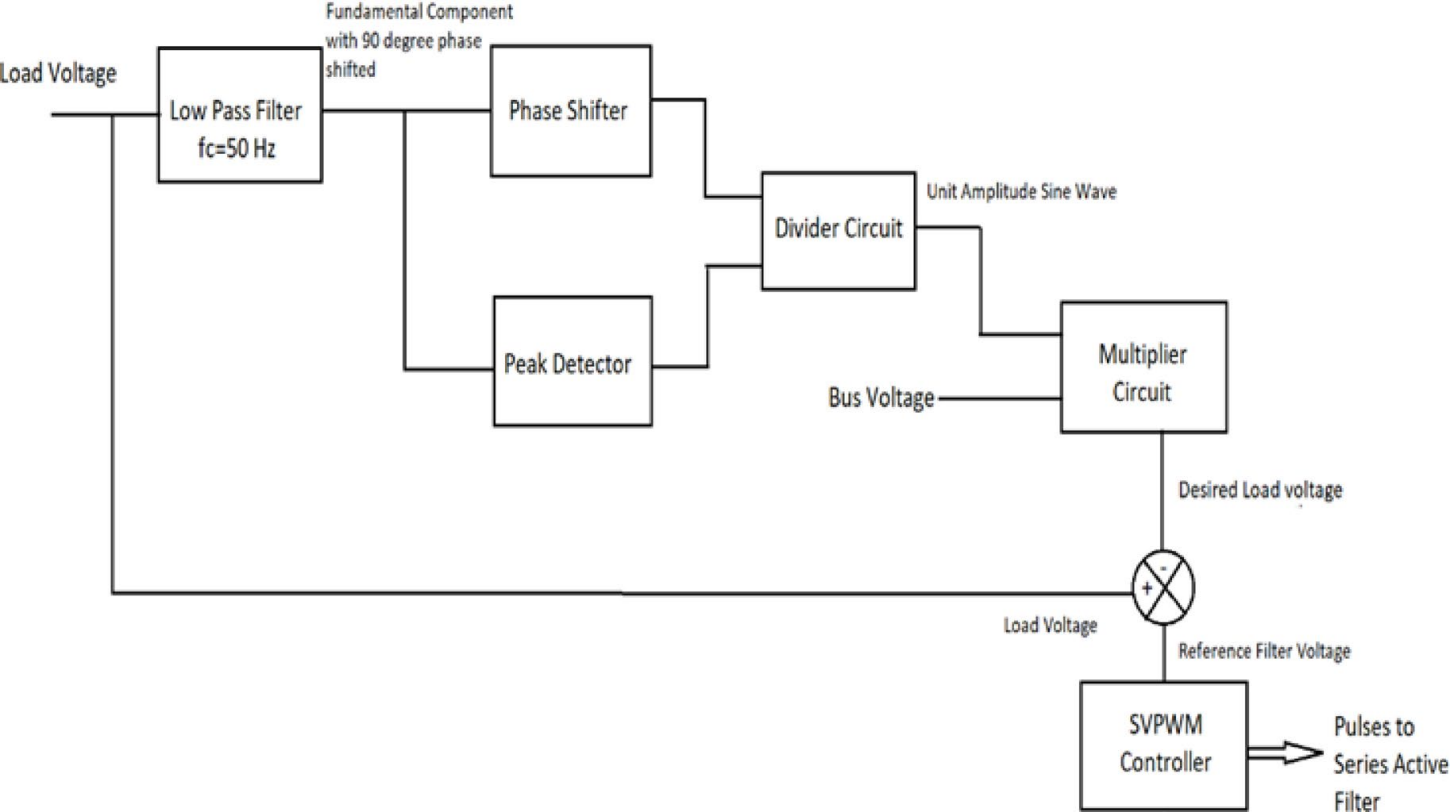
Control algorithm block diagram.

This approach can be used for real-world system conditions such as distorted and asymmetrical ac sources feeding non-linear loads since it requires few calculation steps and straightforward circuits. The active filter algorithm’s fundamental operation is explained by the block diagram in Fig. 6. The Low Pass Filter (LPF) receives the load voltage. The LPF’s output i.e. fundamental component of the load voltage that is 90° phase shifted, is fed into a phase shifter circuit to undo the shift. A peak detector will determine the load voltage fundamental component’s highest amplitude. To obtain the unit amplitude fundamental component sine wave, the output of the phase-shifter circuit is divided by the output of the peak detector. To obtain the necessary load voltage, the bus voltage is multiplied by the unit sine waveform. After deducting the intended load voltage from the actual load voltage, the reference filter voltage is the result. To provide pulses to the two-level inverter, which functions as a series active filter, the reference voltage is supplied to the space vector pulse width modulation controller.

#### Main three-level inverter operation

Two two-level inverters are cascaded to create the three-level inversion^3^. On the second two-level inverter, the top race is attached to the AC point of the first two-level inverter. An isolated dc voltage of Vdc⁄2 is given to both inverters. When both inverters’ top switches are activated, the cascaded inverter’s output voltage is Vdc. Similarly, when the top switch of the second inverter and the bottom switch of the first inverter are on, the output pole voltage takes Vdc⁄2. If the second inverter’s bottom switch is on, the pole voltage also reaches zero. As a result, pole voltage achieves three voltage levels: Vdc, Vdc⁄2, and 0 as indicated in Table 1. The instantaneous values of 3Φ reference voltages are compared in order to identify the sub hexagon center that contains the tip of the reference space vector. To switch the first inverter, this subhex center vector is used. Using the PWM signal, the second inverter is switched.The SVPWM system, as suggested in^35^, is based on the time that the source and load are connected.

#### Supplemental two-level inverter operation

The primary three-level inverter’s voltage harmonic distortions are suppressed by the secondary two-level inverter, which is programmed to function as a series active filter. Minimal computing steps are used in the suggested active filter control technique. The goal of this approach is to minimize motor phase distortions while adhering to IEEE standards. The output voltage of the primary three-level inverter is denoted by V_M_, the output voltage of the secondary two-level inverter by V_F_, and the motor phase voltage by V_O_. The OEWIM system’s motor voltage (V_O_) will be the difference between V_M_ and V_F_. The total of the fundamental component V_1_ and the harmonic component V_n_ will be the primary three-level inverter output V_M_ when it is operated by SVPWM. The harmonic component, or Vn, is the sum of odd, non-triple harmonic order voltages.

1 $$\:{V}_{O}={V}_{M}-{V}_{F}$$

2 $$\:{V}_{M}={V}_{1}+{V}_{n}$$

3 $$\:{V}_{n}={\Sigma\:}{V}_{j}$$

j = odd harmonic order.

The supplemental two-level inverter should be adjusted so that its reference voltage is the difference between V_M_ and V_1_ in order to remove the harmonics of the main inverter.

4 $$\:{V}_{F}={V}_{M}-{V}_{1}={V}_{n}$$

## Results and discussion

### Simulation results

**Fig. 7:**
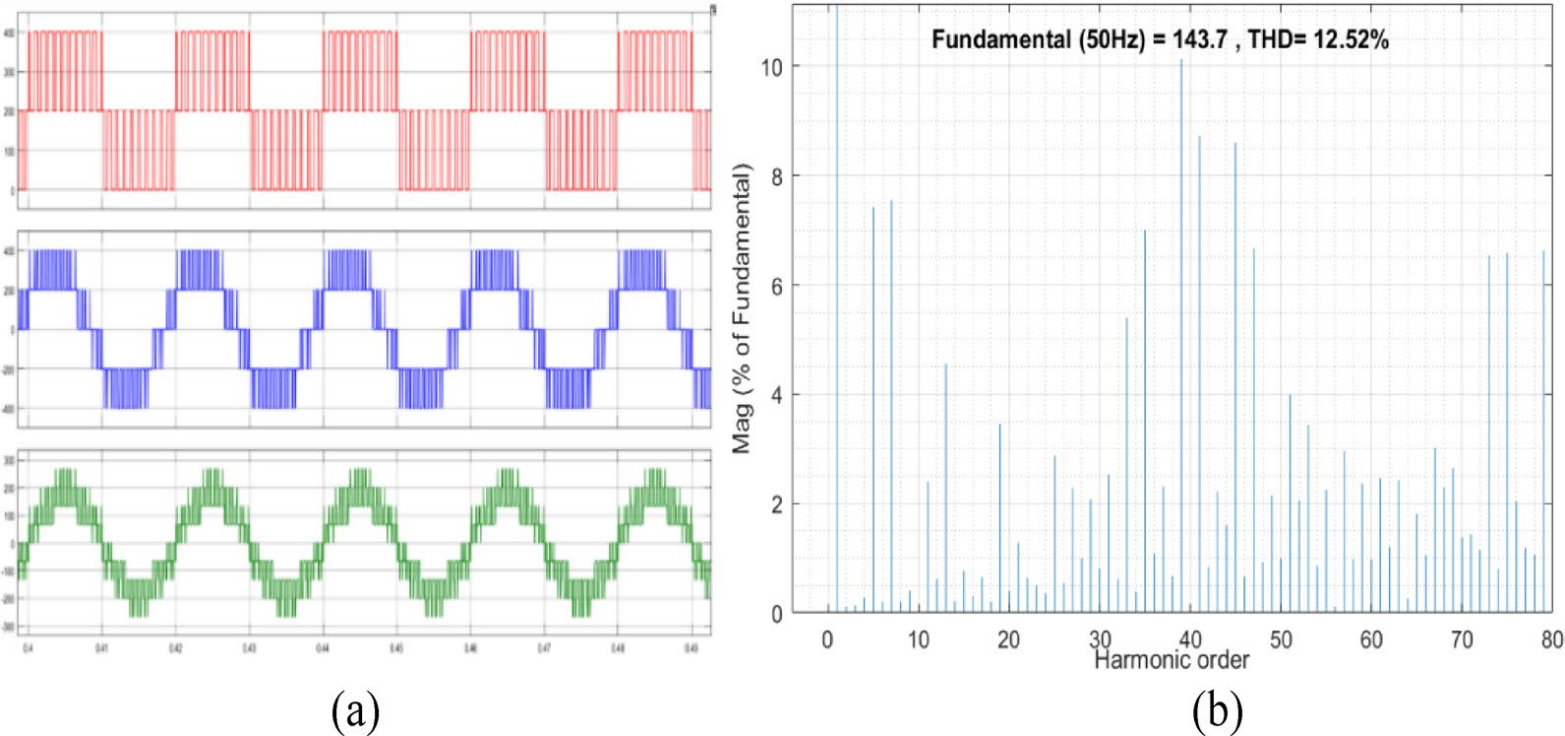
Conventional OEWIM (**a**) Inverter-2 pole voltage, output line voltage and output phase voltage waveforms for m = 0.8 (X label - Time(s), Y label -Voltage (200 V/div), (**b**) FFT analysis of three-level phase voltage.

**Fig. 8:**
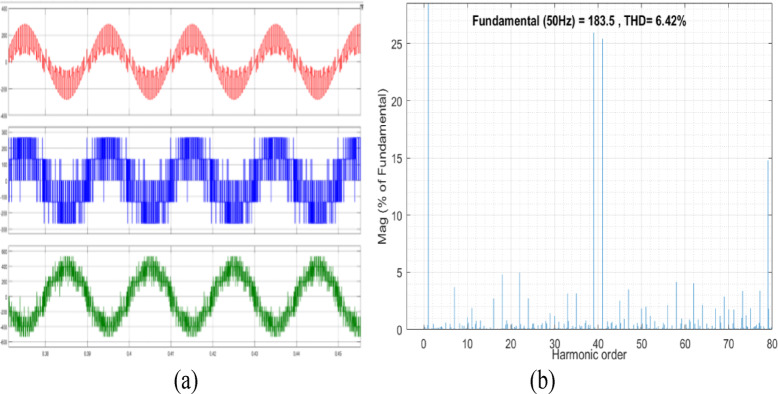
Proposed OEWIM (**a**) Reference filter voltage, actual filter compensating voltage and motor phase voltage (X label - Time(s), Y label -Voltage (200 V/div), (**b**) FFT analysis of motor phase voltage.

The conventional and proposed OEWIM schemes are simulated in MATLAB/Simulink, for a 5HP, 3Φ induction-motor with open loop *v/f* control. The switching frequency of SVPWM controller is 2 kHz, DC link voltage of the VSI is 400 V and modulation index is set as 0.8. The pole voltage of inverter-2, line voltage and phase voltage waveforms with the conventional OEWIM are shown in Fig. 7(a). The FFT analysis of 3- level inverter phase voltage waveform is shown in Fig. 7(b). The THD value of the phase voltage waveform is 12.52%. The suggested system is made up of a 5 HP OEWIM with a series active filter on one side and a PWM fed primary three-level inverter on the other. Figure 8(a) displays the motor phase voltages, the actual filter voltage, and the reference filter voltage. Motor phase voltage THD decreased to 6.42% when the supplemental two-level inverter was used in conjunction with the OEWIM scheme as a series active filter. Figure 8(b) displays the motor phase voltage’s FFT analysis. The comparison between the suggested scheme and the traditional OEWIM setup for the percentage of THD and various harmonic orders with regard to the percentage fundamental magnitude is displayed in Table 2. The outcome confirms that, in comparison to the current design, the suggested method produces fewer harmonic distortions.

In open-end winding induction motor (OEWIM) based multilevel inverter drives, the insertion of dead time plays a crucial role in shaping system performance. Dead time is a short blanking period inserted between the switching of complementary devices in the inverter leg. Dead time, although necessary to avoid shoot-through faults in complementary switching devices, introduces non-idealities in the output voltage waveforms. Its presence results in a reduction of the fundamental component of the applied voltage, accompanied by low-order harmonic distortions, which become particularly severe at low modulation indices and low operating speeds. For OEWIM drives, where two inverters operate simultaneously to synthesize multilevel voltage waveforms, dead time further complicates the interaction between inverter outputs, often causing asymmetry and degrading the quality of staircase voltage synthesis. This distortion propagates into the motor currents, increasing total harmonic distortion (THD), torque ripple, and acoustic noise, while also leading to undesirable common-mode voltage variations and shaft stresses. Introducing a dead time of 2 µs at fs = 2 kHz increases the total harmonic distortion of current from 2.9% to 4.2% at low speed at modulation index m = 0.8.

**Table 2:** Comparison between conventional OEWIM and proposed OEWIM scheme.

Harmonic order	Conventional OEWIM scheme	Proposed OEWIM scheme
5	5.20%	0.28%
7	4.70%	0.81%
9	0.24%	0.01%
11	2.09%	1.67%
13	2.67%	2.36%
15	0.52%	0.11%
17	1.86%	1.79%
19	1.91%	1.60%
%THD	12.52%	6.42%

### Experimental validation

#### Conventional OEWIM scheme

**Fig. 9:**
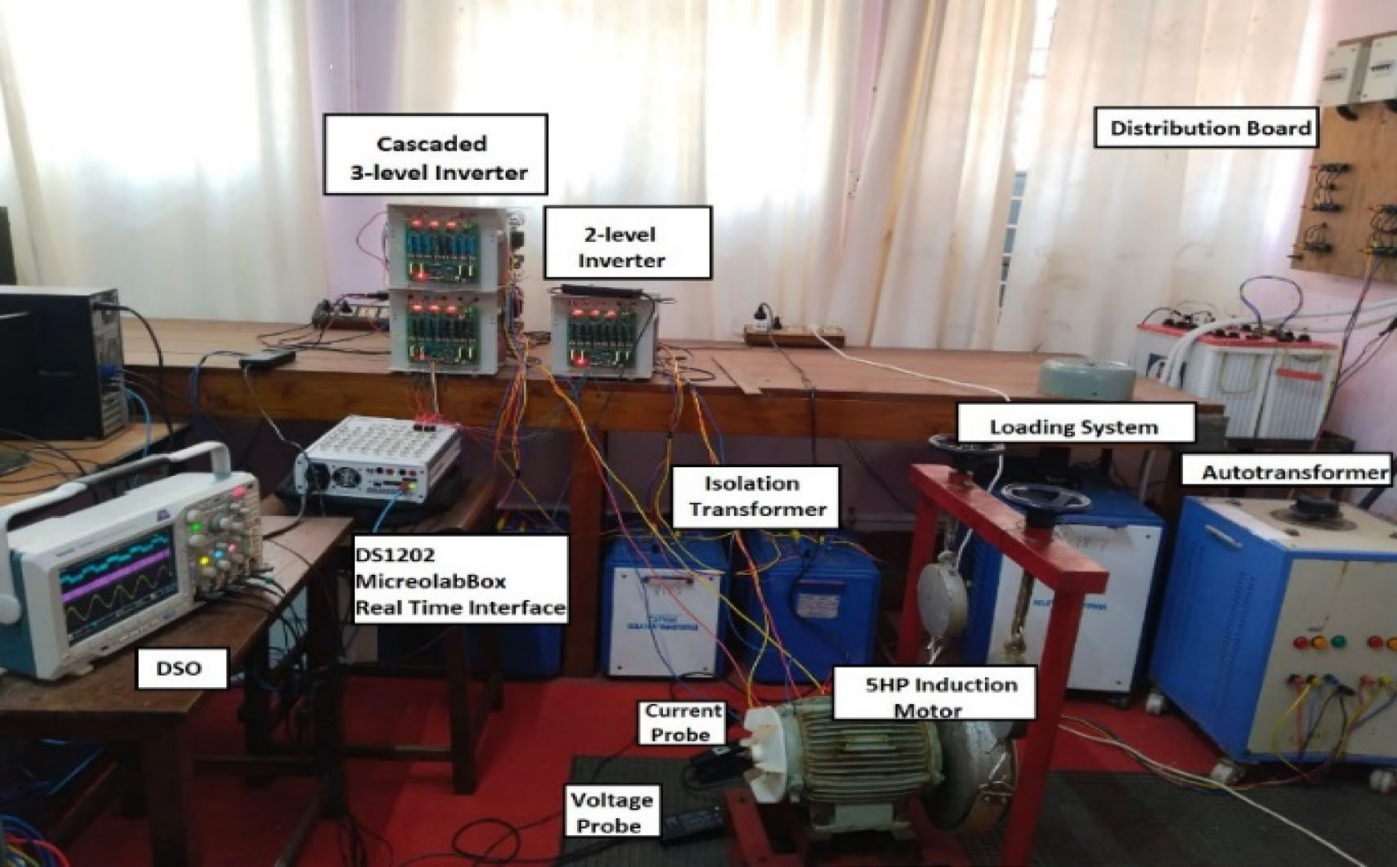
Hardware setup of the proposed scheme.

**Fig. 10:**
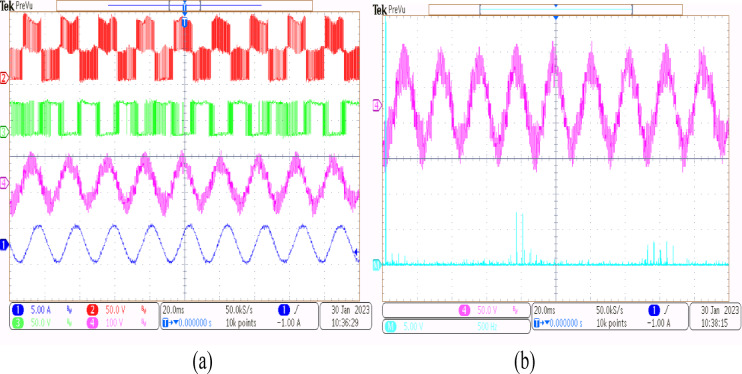
Conventional OEWIM (**a**) Pole voltage of main inverter, pole voltage of supplemental inverter, motor phase voltage and phase current with m = 0.8 (X label - Time(s), Y label -Voltage (50 V/div), Current (5 A/div)) (**b**) FFT analysis of motor phase voltage (50 V/div, 500 Hz/div).

**Fig. 11:**
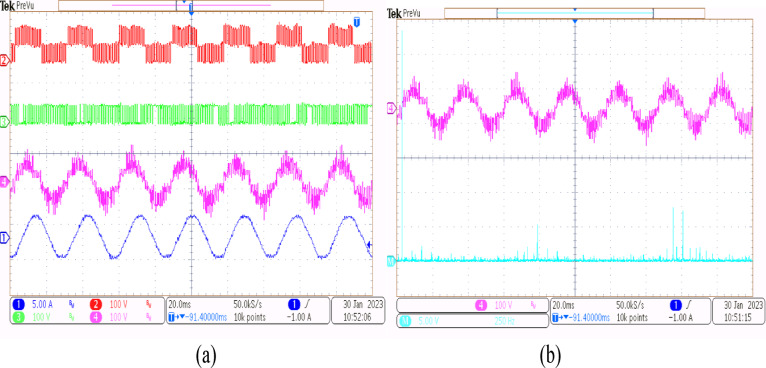
Conventional OEWIM (**a**) Pole voltage of main inverter, pole voltage of supplemental inverter, motor phase voltage and phase current with m = 0.6 (X label - Time(s), Y label -Voltage (100 V/div), Current (5 A/div)) (**b**) FFT analysis of motor phase voltage (100 V/div, 250 Hz/div).

**Fig. 12:**
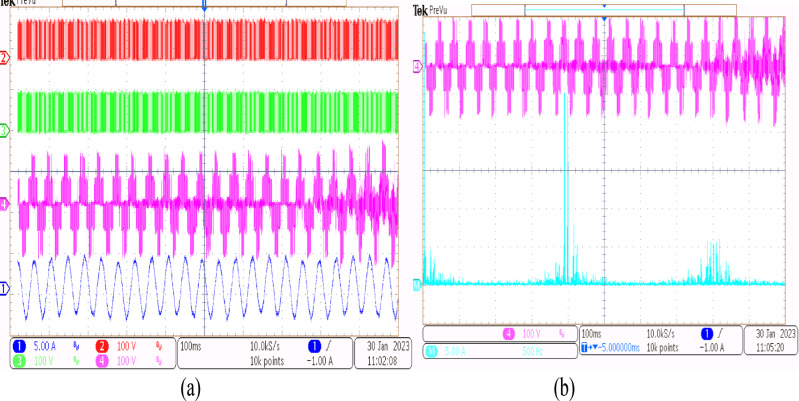
Conventional OEWIM (**a**) Pole voltage of main inverter, pole voltage of supplemental inverter, motor phase voltage and phase current with m = 0.4 (X label - Time(s), Y label -Voltage(100 V/div), Current (5 A/div)) (**b**) FFT analysis of motor phase voltage (100 V/div, 500 Hz/div).

**Fig. 13:**
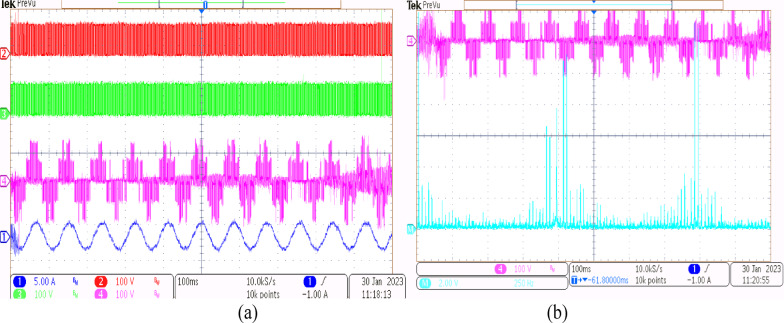
Conventional OEWIM (**a**) Pole voltage of main inverter, pole voltage of supplemental inverter, motor phase voltage and phase current with m = 0.2 (X label - Time(s), Y label -Voltage(100 V/div), Current(5 A/div)) (**b**) FFT analysis of motor phase voltage (100 V/div, 250 Hz/div).

**Fig. 14:**
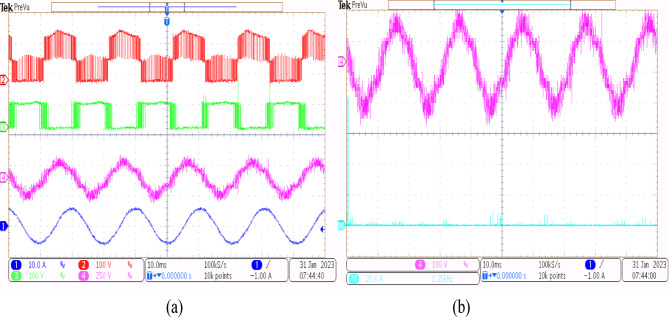
Conventional OEWIM (**a**) Pole voltage of main inverter, pole voltage of the supplemental inverter, motor phase voltage and phase current with m = 1.1 (X label - Time(s), Y label -Voltage (100 V/div), Current (10 A/div)) (**b**) FFT analysis of motor phase voltage (100 V/div, 1250 Hz/div).

Using the conventional SVPWM-based OEWIM system, a 5-HP, 415 V, 1430 rpm induction motor was driven by an 11.5 kVA two-level inverter with open loop *V/f* control. Figure 9 depicts the experimental configuration. The dSpace DS1104 RTI platform and a sample frequency of 20 kHz were used to accomplish the suggested technique. Figure 10(a) shows the main inverter pole voltage, supplemental inverter pole voltage, motor phase voltage and phase current for modulation index, m = 0.8, for the traditional OEWIM method. Figure 10(b) displays the motor phase voltage FFT analysis, which shows high percentage of lower order harmonic content and total harmonic distortion value of 14.3%. The higher order harmonics are found scattered over the sidebands of switching frequency. The motor phase voltage, phase current, supplemental inverter, and main inverter pole voltages are displayed in Fig. 11(a) for the modulation index, m = 0.6. Figure 11(b) shows the FFT analysis of the resulting phase voltage waveform with a voltage THD value of 15.8%. Figure 12(a) and Fig. 12(b) show the motor phase voltage and phase current waveforms with m = 0.4 as well as the phase voltage FFT spectrum with a THD of 17.2%. Figure 13(a) and Fig. 13(b) show the motor phase voltage and phase current waveforms with m = 0.2 as well as the phase voltage FFT spectrum. The pole voltage of main inverter, pole voltage of the supplemental inverter, motor phase voltage and phase current with modulation index, m = 1.1 for the conventional OEWIM scheme is given in Fig. 14(a). The FFT analysis of corresponding motor phase voltage is given in Fig. 14(b). On comparing the FFT spectrum of phase voltage with different modulation levels, the total harmonic distortion value of phase voltage is found to be much higher than the IEEE 519 standard value. Also, the distortion are found to be higher for lower modulation index values. For overmodulation (m = 1.1), there is less switching of inverter switches.

#### Proposed OEWIM scheme

**Fig. 15:**
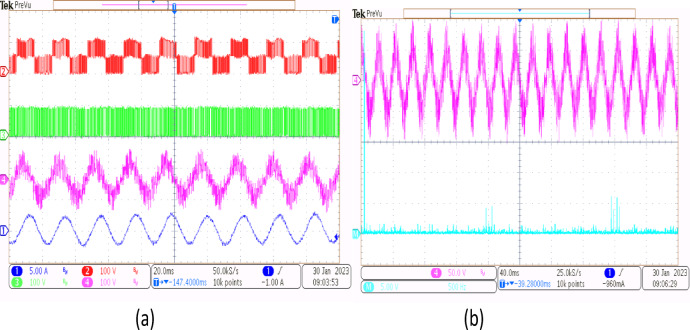
Proposed OEWIM (**a**) Pole voltage of main inverter, pole voltage of supplemental inverter, motor phase voltage and phase current with m = 0.8 (X label - Time(s), Y label -Voltage (100 V/div) Current (5 A/div)) (**b**) FFT analysis of motor phase voltage (50 V/div, 500 Hz/div).

**Fig. 16:**
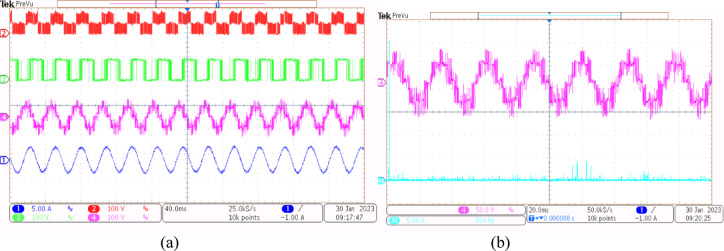
Proposed OEWIM (**a**) Pole voltage of main inverter, pole voltage of supplemental inverter, motor phase voltage and phase current with = 0.6 (X label - Time(s), Y label -Voltage (100 V/div), Current (5 A/div)) (**b**) FFT analysis of motor phase voltage (50 V/div, 500 Hz/div).

**Fig. 17:**
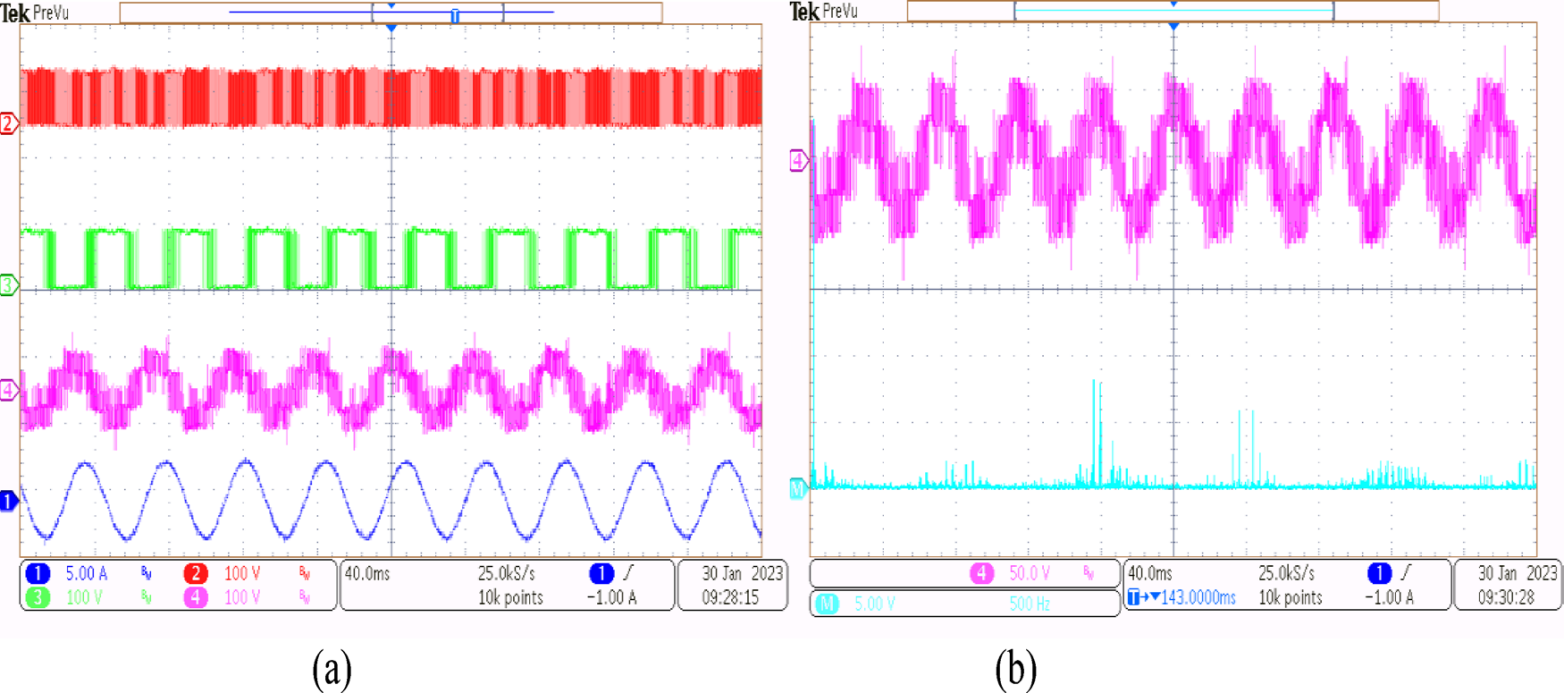
Proposed OEWIM (**a**) Pole voltage of main inverter, pole voltage of supplemental inverter, motor phase voltage and phase current with m = 0.4 (X label - Time(s), Y label -Voltage (100 V/div), Current (5 A/div)) (**b**) FFT analysis of motor phase voltage (50 V/div, 500 Hz/div).

**Fig. 18:**
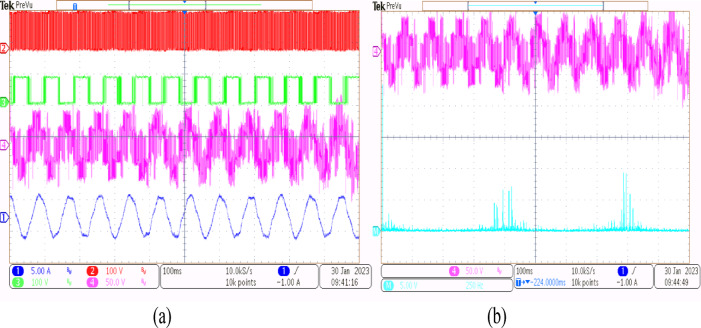
Proposed OEWIM (**a**) Pole voltage of main inverter, pole voltage of supplemental inverter, motor phase voltage and phase current with m = 0.2 (X label - Time(s), Y label -Voltage (100 V/div), Current (5 A/div)) (**b**) FFT analysis of motor phase voltage (50 V/div, 250 Hz/div).

**Fig. 19:**
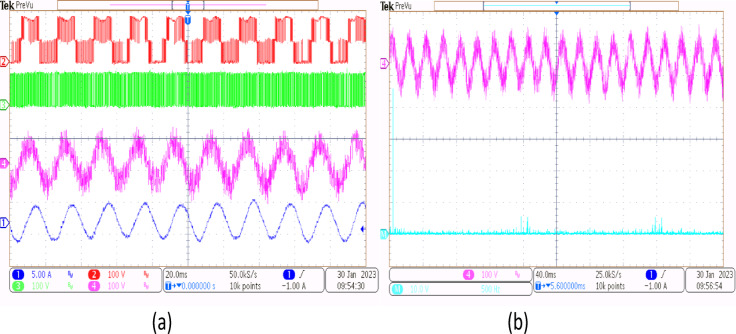
Proposed OEWIM (**a**) Pole voltage of main inverter, pole voltage of supplemental inverter, motor phase voltage and phase current with m = 1.1 (X label - Time(s), Y label -Voltage (100 V/div), Current (5 A/div)) (**b**) FFT analysis of motor phase voltage (100 V/div, 500 Hz/div).

**Fig. 20:**
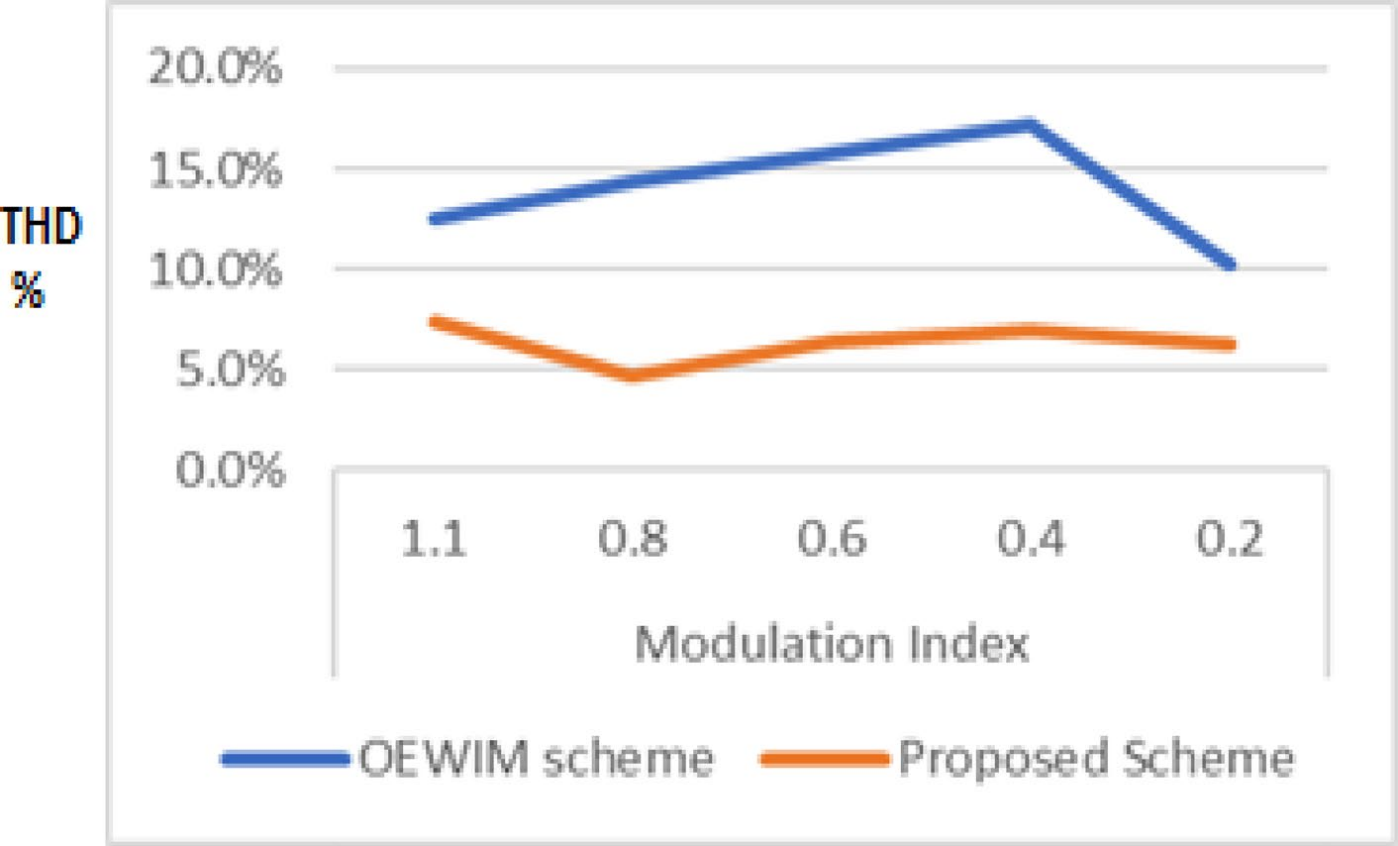
Comparison between conventional OEWIM and proposed OEWIM scheme based on experimental.

A 5-HP, 415 V, 1430 rpm induction motor with mechanical loading was used to test the suggested SVPWM-based active filter control of dual inverter-fed OEWIM scheme, as shown in Fig. 4. The proposed system was validated for different modulation indices. The dSpace DS1104 RTI platform, which has a sampling frequency of 20 kHz, is used to implement the proposed schemes. Figure 15(a) displays the pole voltage of the main inverter, pole voltage of the supplemental inverter, motor phase voltage and phase current with m = 0.8 of the proposed scheme. FFT analysis of the resultant motor phase voltage is shown in Fig. 15(b). The voltage total harmonic distortion value of the phase voltage is 4.6% for the proposed OEWIM. Also, the FFT spectrum shows less lower order harmonic distortion as compared with the conventional OEWIM scheme.The pole voltage of the main inverter, pole voltage of the supplemental inverter, motor phase voltage and phase current with m = 0.6 of the proposed scheme are illustrated in Fig. 16(a). The harmonic spectrum of the motor phase voltage is shown in Fig. 16(b), which gives a total harmonic distortion value of 6.4%. The pole voltages, resultant motor phase voltage, phase current waveforms and motor phase FFT spectrum for modulation indices m = 0.4, 0. 2 and 1.1 are depicted in Figs. 17, 18 and 19 respectively. Table 3; Fig. 20 illustrates the comparison between conventional OEWIM and the proposed OEWIM scheme based on experimental results. For all modulation indices, the obtained findings verify that the suggested method produced lower phase voltage distortions than the standard scheme. Also lower harmonics are much less in proposed OEWIM scheme as compared to conventional OEWIM.

**Table 3:** Comparison between conventional OEWIM and proposed OEWIM scheme.

Modulation index	Conventional OEWIM scheme	Proposed OEWIM scheme
1.1	12.5%	7.4%
0.8	14.3%	4.6%
0.6	15.8%	6.4%
0.4	17.2%	7.0%
0.2	10.3%	6.2%

## Conclusion

To reduce harmonic distortions in a three-level inverter, an open-end winding induction motor scheme is proposed. By cascading two two-level inverters together, a three-level primary inverter is produced. A secondary two-level inverter improves power quality, while a primary three-level inverter provides the necessary active power compared to a traditional open-end winding motor drive. The secondary two-level inverter uses the series active filtering control approach, while the primary three-level inverter operates via SVPWM control. In order to eliminate harmonic distortions in the motor phase voltages, the active filtering control method is adopted which involves less computational steps. The suggested method provided smaller phase voltage distortions than the conventional scheme, which is validated by the results of simulations. The proposed scheme is experimentally validated for a loaded 5 HP induction motor. Experimental measurements show that the proposed SVPWM series compensation achieved a THD reduction of ~ 48% compared to conventional SVPWM fed OEWIM. These systems effectively mitigate harmonic voltage distortions and support higher levels of modularity and fault-tolerant operations. Furthermore, validation was carried out only under balanced load and standard test conditions, performance under unbalanced supply or fault-tolerant operation remains unexplored. Future work will address scalability, real-time implementation under varying industrial loads, and comparative cost-benefit analysis.

## Data Availability

No datasets were generated or analysed during the current study.
